# A Neural-Network-Free Calibration Matches or Beats Deep Single-Cell Perturbation Response Models Across Four Datasets

**DOI:** 10.3390/genes17070816

**Published:** 2026-07-17

**Authors:** Bingchi Sun, Haibin Zheng, Jinyin Chen, Jingjing Jin

**Affiliations:** 1 Zhejiang University of Technology, Hangzhou 310023, China; peter05090806@163.com (B.S.); chenjinyin@zjut.edu.cn (J.C.); 2 Key Laboratory of Beijing Life Science Academy, Beijing 102206, China; jinjj@ztri.com.cn; 3 Key Laboratory of Data Protection and Intelligent Management, Ministry of Education, Sichuan University, Chengdu 610065, China

**Keywords:** single-cell RNA-seq, perturbation response prediction, leave-one-out generalisation, distribution calibration, affine moment matching, benchmarking

## Abstract

**Background/Objectives**: Deep generative models such as scGen are the standard for predicting how an unseen cell type or species responds to a perturbation under leave-one-group-out (LOCO). We ask whether the deep model’s advantage over a one-line linear baseline is learnable without any neural network. **Methods**: We decompose scGen’s edge into two leakage-free analytic pieces (per-type response magnitude and per-gene response direction) and add a per-gene affine moment-matching step. The resulting calibration, AMM-SimWMag, uses no neural network and is CPU-only. We evaluate it on a nine-metric panel across 4 datasets spanning 3 biologies and 2 LOCO axes (cell type and species), using a dataset-stratified test (a Stouffer combination of per-dataset signed-rank tests, Holm-adjusted). **Results**: AMM-SimWMag is competitive-or-best (within 0.003 of scGen) on 8–9 of 9 metrics on each of 4 datasets. It significantly improves on scGen on eight of nine metrics, favoured in all four datasets on seven; the lone exception, the per-gene log-fold-change correlation, is a tie-or-better everywhere. Against three modern baselines (scPRAM, biolord and CPA) on the same protocol, it beats biolord and CPA on 9/9 metrics and scPRAM on 7/9 (pooled). The affine moment-matching step improves distribution distances (MMD, energy, sliced-Wasserstein), though C2ST stays near 1.0 for all methods. **Conclusions**: AMM-SimWMag recovers the deep model’s advantage over a linear baseline without any neural network. No single deep model is robust across all four biologies, whereas AMM-SimWMag matches or beats them across 4 datasets, CPU-only and reproducibly.

## 1. Introduction

Predicting how individual cells respond to a perturbation—an interferon, a pathogen, or a drug—is a core goal of functional genomics and a building block of the emerging “virtual cell” programme, which aims to simulate cellular behaviour across states directly from data [1]. Reliable in silico response prediction would let researchers prioritise experiments, reduce the combinatorial cost of profiling every cell type under every condition, and generate testable hypotheses about gene regulation. The biology that anchors our benchmarks is itself instructive. The type-I interferon (IFN-β) response is a tightly regulated, well-characterised transcriptional programme in which interferon-stimulated genes are induced to markedly different degrees across immune cell types [2], and the innate immune response to stimulation is broadly conserved yet quantitatively divergent across mammalian species [3]. A model that transfers responses across cell types or species must therefore capture both a *conserved direction* of the response and a *type- or species-specific magnitude*—a structure we exploit directly below.

A central promise of single-cell generative modelling is *perturbation transfer:* given the response of several cell types to a treatment, it predicts the response of a held-out type (or, more ambitiously, a held-out species). scGen [4] formalised this as latent vector arithmetic in a variational autoencoder (VAE) [5] and is the standard deep baseline; a long line of work [6,7] extends the idea. The standard evaluation is leave-one-group-out (LOCO): train on all but one group, predict that group’s stimulated state from its control cells plus the other groups’ responses, and score the predicted against the real stimulated cells.

A recurring, uncomfortable observation is that a *one-line* “linear delta” baseline, which simply adds the mean control→stimulated shift of the other groups to the held-out group’s control mean, is already close to scGen on the headline metric. This raises a sharp question: *is scGen’s residual advantage learnable without a neural network at all?* If the gap decomposes into a small number of leakage-free, analytically computable quantities, then the right contribution is not a bigger model but a better *calibration*.

This paper answers the question in the affirmative. Our contributions are:

1. **A decomposition of the deep model’s edge.** Cheap, CPU-only diagnostics (Section 4) localise scGen’s advantage over the linear baseline to exactly two leakage-free factors: per-type response *magnitude* and per-gene response *direction.* Both are recovered analytically.
2. **A neural-network-free calibration,** **AMM-SimWMag** (*Affine Moment Matching + Similarity-Weighted Magnitude)* (Section 5): per-type magnitude × similarity-weighted direction for the mean, plus per-gene affine moment matching for the distribution. It uses no learned model and no held-out stimulated data.
3. **Comprehensive, multi-dataset, multi-axis validation** (Section 6): competitive-or-best vs. scGen on 8–9/9 metrics on *4* datasets across 2 LOCO axes; statistically significant on 8/9 metrics under a dataset-stratified test (a Stouffer combination over the four datasets), corroborated by the pooled test. We further benchmark three *modern* published models (scPRAM [8], biolord [9], and CPA [7]) on the same protocol (Section 6.5): none is robust across all four biologies, and the calibration is significantly better than each pooled.
4. **Two honest negative results:** C2ST saturates near 1.0 for every method evaluated here (and for oracle moment- and hurdle-based approximations), so it does not separate methods on these data, and the per-gene log-fold-change correlation is a tie (not a significant win) once tested rigorously. We report both to avoid cherry-picking.

## 2. Related Work

**Predicting Perturbation Responses.** Generative and representation learning models dominate single-cell perturbation prediction [10]. scGen [4] performs latent vector arithmetic in a VAE; scVI [6] provides the probabilistic latent space such methods build on; CPA [7] and its chemical extension chemCPA [11] disentangle perturbation, dose and covariate factors for combinatorial and dose screens; CellOT [12] learns a neural optimal-transport map between unperturbed and perturbed distributions (optimal transport is now a broad framework for single-cell mapping and alignment [13]); and more recent models add attention and optimal transport over a VAE (scPRAM [8]) or explicit latent disentanglement (biolord [9]). GEARS [14] instead predicts *unseen genetic* perturbations from a gene–gene knowledge graph, a related but distinct task. We benchmark scGen, scPRAM, biolord and CPA directly because they target the same single-stimulus, unseen-group transfer that we study.

**Single-cell foundation models.** A parallel line pretrains to large transformers on tens of millions of cells (scBERT [15], Geneformer [16], scGPT [17], scFoundation [18] and CellPLM [19]; reviewed in [20]) and offers perturbation response prediction as one downstream task. These models are powerful but expensive, and their advantage over simple predictors on perturbation tasks is contested (below). Our calibration is at the opposite end of the spectrum: no pretraining, no neural network, and CPU-only.

**Critical benchmarking and simple baselines.** A growing body of work cautions that strong deep models often fail to beat deliberately simple baselines on perturbation prediction. Most directly, Ahlmann-Eltze, Huber and Anders [21] show that five foundation models and two other deep models do not consistently outperform a linear or mean baseline for genetic perturbation effects. This pattern is corroborated by independent benchmarks: zero-shot foundation-model embeddings do not consistently improve perturbation effect prediction over simple baselines, especially under distribution shift [22], and a systematic comparison of twelve methods across many datasets finds that fine-tuned foundation models share a conservative bias that compresses response variance [23]. Our results are in the same spirit but address a different question: rather than only *reporting* that a linear baseline is competitive, we *decompose* the deep model’s residual edge into leakage-free analytic factors and show it can be recovered without any network.

**Data resources and evaluation.** Public perturbation atlases [24,25,26,27] and harmonised collections such as scPerturb [28] have made systematic benchmarking possible; scPerturb also popularised the energy distance (E-distance) as a perturbation effect measure, one of the nine metrics we report. We score distributional agreement with the maximum mean discrepancy [29], energy distance [30] and sliced-Wasserstein distance [31], and separability with a classifier two-sample test [32].

**Novelty relative to prior methods.** AMM-SimWMag differs from the models above in kind, not only in degree. scGen [4], scPRAM [8], biolord [9] and CPA [7] all learn a neural latent space in which the perturbation is applied, and CellOT [12] and moscot [13] learn a neural optimal-transport map; foundation models [15,16,17,18,19] add large-scale pretraining. In contrast, our method contains no neural network, no pretraining and no iterative optimisation: it is a closed-form calibration that (i) makes explicit the two quantities we show carry scGen’s edge—a per-type response *magnitude* and a control-only similarity-weighted *direction,* both estimated analytically and leakage-free—and (ii) applies a per-gene affine moment-matching transport that is provably exact on the first two moments and preserves gene–gene correlation (Proposition 1). Relative to the linear-delta baseline, it is not merely “linear plus tuning”: it adds a leakage-free per-type magnitude scalar, a similarity-weighted direction, and a second-moment transport, none of which the linear baseline has. The contribution is therefore a transparent, inspectable and CPU-only *explanation and reconstruction* of where a deep model’s advantage comes from, rather than another black-box predictor.

## 3. Materials and Methods: Datasets, Protocol, and Metrics

**Datasets.** We use four publicly available scRNA-seq perturbation datasets spanning three biologies. (i) The *Kang* IFN-β peripheral-blood mononuclear cell (PBMC) dataset [33]: seven immune cell types (B, CD4 T, CD8 T, NK, CD14^+^ monocytes, FCGR3A^+^ monocytes, and dendritic cells), control vs. IFN-β stimulation, provided as frozen log-normalised expression. (ii, iii) Two intestinal-infection arms built from the *Haber* small-intestinal atlas [34]: Control vs. *Salmonella* (5010 cells) and Control vs. *H. polygyrus* day 10 (5951 cells), each with eight epithelial cell types (stem, transit-amplifying and early transit-amplifying, enterocyte and enterocyte progenitor, goblet, tuft, and enteroendocrine), holding out one cell type at a time. (iv) The *Hagai* cross-species dataset [35]: bone marrow-derived phagocytes, unstimulated vs. LPS 6 h, across four species (rat, rabbit, mouse, pig), holding out a whole species (77,642 cells × 6000 genes). Unless otherwise noted, expression is top 6000 highly variable gene (HVG) log1p(CP10k) with raw counts retained where available.

**Protocol.** All experiments use leave-one-group-out (LOCO). For a held-out group *h* we use (i) the other groups’ control and stimulated cells and (ii) the held-out group’s *control* cells; we predict *h*’s stimulated cells and score against the real held-out stimulated cells, which are used *only for scoring,* identically for every method. We average over three seeds and report the mean over held-out groups. Data are log-normalised HVG expression following standard practice [36] (Scanpy [37]); scGen follows the authors’ protocol (150 VAE epochs) and reproduces the frozen benchmark numbers byte-for-byte, so all comparisons are apples-to-apples. By “frozen benchmark” we mean our archived scGen reference run—scGen trained once under the authors’ protocol at fixed seeds, with its per-fold predictions frozen and provided with the code (Section 9)—against which every method is then scored by the identical pipeline; we re-load these frozen outputs rather than retraining scGen per comparison, so the reported numbers are exactly reproducible.

**Leakage audit.** Every method uses only *other* groups’ stimulated cells; the held-out group’s control profile is used only for magnitude prediction, direction weighting, and the affine map—*never* its stimulated cells. This audit is preserved across all methods and datasets. The same held-out control cells are supplied as input to *every* method—scGen and the modern deep baselines encode them to seed generation—so AMM-SimWMag’s use of them as the base population for the affine transport confers no extra access: the held-out control population is part of the LOCO inputs given identically to all methods, and only the held-out *stimulated* cells are withheld for scoring.

**Metrics (9).** We score the mean, the variance, the per-gene direction, and the full distribution: R^2^ of the predicted vs. real per-gene mean over all genes (**R^2^-mean**) and over the top 100 differentially expressed genes (**R^2^-DEG)**; R^2^ of the per-gene *variance* over all genes and the top DEGs (**R^2^-var**, **R^2^-var-DEG**); the Pearson correlation of the per-gene log-fold-change (**deg_lfc_r**); and three distribution distances—maximum mean discrepancy (**MMD**) [29], **energy** distance [30], and **sliced-Wasserstein** [31]—plus a classifier two-sample test AUC (**C2ST**) [32] (a RandomForest on 50 PCs; 0.5 = indistinguishable, 1.0 = perfectly separable). Higher is better for the R^2^ metrics and deg_lfc_r; lower is better for MMD, energy, sliced-W, and C2ST. The C2ST classifier is specified in full for reproducibility: real held-out stimulated cells and generated cells are balanced to $n=\min(n_{\mathrm{real}},n_{\mathrm{gen}})$ by random subsampling, reduced to the top 50 principal components (PCA fit on the pooled 2n cells), and separated by a random forest (200 trees, maximum depth 8); the reported AUC is the mean over stratified 5-fold cross-validation, so every cell serves once in a test fold and no separate calibration split is held out. The **R^2^-DEG** and **R^2^-var-DEG** metrics restrict the score to the top-100 genes ranked by the *real* held-out response (mean stimulated − mean control); this ranking is used only to define the evaluation subset, never as an input to any prediction, and the held-out stimulated cells are used only for scoring (Proposition 2). The reported C2ST AUCs are read from the frozen per-fold prediction panels rather than by re-training the classifier, and re-running the scorer across seeds 0,1,2 leaves them essentially unchanged (per-method cross-seed SD ≤0.0065), so the near-1.0 saturation is not a seed artefact.

## 4. Diagnostics: Where Is the Deep Model’s Edge?

We ran a chain of cheap, CPU-only diagnostics *before* committing any GPU time, to localise scGen’s advantage on the Kang IFN-β dataset [33].

**(1) The mean’s only learnable gap is per-type*****magnitude*****.** The IFN-β shift is dominated by a *shared* direction; a single global delta under-shoots strong responders (monocytes, dendritic cells) and over-shoots weak ones. An oracle per-type scalar lifts R^2^-DEG 0.810→0.877, and a single *leakage-free* feature—baseline interferon-stimulated-gene expression, computed from other types only—recovers it: R^2^-DEG 0.872≈ oracle, matching scGen. So scGen’s edge over the linear baseline on the mean is *just* magnitude calibration.

**(2) Variance is shared and predictable.** The per-gene control→stim variance change is partly shared across types: $\mathrm{var}\_\mathrm{pred}=\mathrm{control}\_\mathrm{var}+{\mathrm{mean}}_{\mathrm{other}}\left(\mathrm{stim}\_\mathrm{var}-\mathrm{control}\_\mathrm{var}\right)$ predicts the held-out stim variance at $R^{2}\approx 0.78$, far above every method in the benchmark (linear/control-as-pred 0.65, scGen 0.60). Here control_var is the held-out group’s control variance and ${\mathrm{mean}}_{\mathrm{other}}\left(\cdot \right)$ averages the variance change over the other (training) types t≠h; var_pred is reused as the target variance in Section 5(b). 

**(3) C2ST is saturated for all evaluated methods (negative result).** Every method scores C2ST ≈1.0. A four-step diagnostic shows why: even a Gaussian drawn from the *true* held-out mean and covariance scores 0.998 and an oracle hurdle model with *true* sparsity and marginals reaches only 0.944, because the data are 95.3% exact zeros, non-Gaussian, and gene-correlated. Across the methods and oracle approximations we evaluated, none brings C2ST below this near-1.0 ceiling under LOCO; we therefore report C2ST as *saturated* for these generators rather than claiming a proof that no generator could ever win. We report this as an explanation, not a modelling failure.

## 5. Method: **AMM-SimWMag**

The calibration has three leakage-free parts and *no neural network*.

**Problem and notation.** Fix a held-out group *h*. Let $C_{h}\in R^{n_{h}\times G}$ be its control cells over *G* genes, with per-gene mean and variance $\mu_{h}^{c},v_{h}^{c}\in R^{G}$ (so $\mu_{h,g}^{c}\equiv \mathrm{ctrl}\_{\mathrm{mean}}_{g}$ and $v_{h,g}^{c}\equiv \mathrm{ctrl}\_{\mathrm{var}}_{g}$); the real stimulated cells $S_{h}$ are *withheld* and used only for scoring. For every other group t≠h the control and stimulated cells—hence $\mu_{t}^{c}$ and the response delta $\delta_{t}=\mu_{t}^{s}-\mu_{t}^{c}$—are available. The task is to build a predicted stimulated population ${\hat{S}}_{h}$ from the *LOCO-admissible* inputs $A_{h}=\left\{C_{h}\right\}\cup {\left\{\left(\mu_{t}^{c},\delta_{t}\right)\right\}}_{t\neq h}$ alone, so that its distribution matches that of $S_{h}$. We factor this into predicting the per-gene mean ${\hat{\mu }}_{h}$, the per-gene variance ${\hat{v}}_{h}$, and a per-cell transport that realises both; the three parts below estimate these in turn.

**(a) Magnitude calibration of the mean.** We let $\mathrm{ctrl}\_{\mathrm{mean}}_{h}$ be the held-out group’s per-gene control mean and let global_delta be the control→stimulated mean shift obtained by pooling all *other* (training) types’ cells. We scale this shared delta by a per-type scalar $\hat{s}$:

(1) $$\mathrm{mean}\_\mathrm{pred}=\mathrm{ctrl}\_{\mathrm{mean}}_{h}+\hat{s}\cdot \mathrm{global}\_\mathrm{delta}.$$

We obtain $\hat{s}$ leak-free: over the other types we least-squares fit a line from one scalar feature—each type’s mean baseline expression over a leak-free interferon-stimulated-gene (ISG) set (top 200 response genes from other types)—to that type’s magnitude scalar (its delta projected onto global_delta over its top-100 DEGs), evaluate it at the held type, and clip to [0.3,2.5]. No held-type stimulated cells are used.

**(a′) Similarity-weighted direction.** A single global delta cannot match a held type whose response points differently from the population average—exactly what deg_lfc_r measures. We replace it with a control-only (hence leak-free) similarity-weighted kernel average of the other types’ deltas. For each other type t≠h we let $d_{t}={∥\mathrm{ctrl}\_{\mathrm{mean}}_{h}-\mathrm{ctrl}\_{\mathrm{mean}}_{t}∥}_{2}$ be the Euclidean distance between baseline (control) profiles and let $\tilde{d}={\mathrm{median}}_{t\neq h}\;d_{t}$ be the median of those distances (a bandwidth); then

(2) $$\begin{array}{cc} \mathrm{sw}\_w_{t} & =\frac{\exp(-d_{t}/\tilde{d})}{\sum_{t^{\prime }\neq h}\exp\left(-d_{t^{\prime }}/\tilde{d}\right)}\;\left(a\;\mathrm{softmax}\;\mathrm{over}\;t\neq h\right), \end{array}$$

(3) $$\begin{array}{cc} \mathrm{simw}\_\mathrm{delta} & =\sum_{t\neq h}\mathrm{sw}\_w_{t}\;\left(\mathrm{stim}\_{\mathrm{mean}}_{t}-\mathrm{ctrl}\_{\mathrm{mean}}_{t}\right), \end{array}$$

(4) $$\begin{array}{cc} \mathrm{mean}\_\mathrm{pred} & =\mathrm{ctrl}\_{\mathrm{mean}}_{h}+{\hat{s}}_{\mathrm{sw}}\cdot \mathrm{simw}\_\mathrm{delta}, \end{array}$$

where the weights are non-negative and sum to one, and ${\hat{s}}_{\mathrm{sw}}$ is the part-(a) magnitude scalar refit with simw_delta replacing global_delta. Weights use control profiles only and deltas use *other* types only, so there is no leakage.

**(b) Affine moment matching (the distribution).** We generate held-out stimulated cells by per-gene affine-rescaling the *real* held-out control cells to the predicted (mean, variance); matching first and second moments by an affine map is the single-gene analogue of correlation-alignment domain adaptation [38]:

(5) $$x_{\mathrm{pred}}\left[:,g\right]={\mathrm{mean}}_{g}+\left(x_{\mathrm{ctrl}}\left[:,g\right]-\mathrm{ctrl}\_{\mathrm{mean}}_{g}\right)\cdot \sqrt{{\mathrm{var}}_{g}/\mathrm{ctrl}\_{\mathrm{var}}_{g}}.$$

Here *g* indexes genes; ${\mathrm{mean}}_{g}$ is the predicted mean from (a′) (Equation (4)); ${\mathrm{var}}_{g}$ is the shared-variance prediction var_pred of Diagnostic (2); $\mathrm{ctrl}\_{\mathrm{mean}}_{g},\mathrm{ctrl}\_{\mathrm{var}}_{g}$ are the held-out control mean and variance; and $x_{\mathrm{ctrl}}\left[:,g\right]$ are the real held-out control cells, resampled with replacement. Both variances are floored at $\varepsilon =10^{-6}$ so the rescaling stays defined for all-zero genes (∼95% of entries are zero). The map is *exact* on the mean and variance and preserves the control cells’ gene–gene correlation; it uses no held-out stimulated data. The affine map itself is *deterministic*—the only randomness is which control cells are resampled—so AMM-SimWMag transports the control population rather than sampling anew, adding no stochasticity beyond the control cells (revisited in Section 7).

**Properties.** Two properties explain why an analytic calibration with no learned model wins the distribution *distances,* and a third bounds its cost.

**Proposition** **1** (Moment exactness and correlation preservation)**.** *For each gene g the affine map of Equation (5) sends the held-out control cells to a population whose per-gene mean is exactly ${\mathrm{mean}}_{g}$ and whose per-gene variance is exactly ${\mathrm{var}}_{g}$, and it leaves the Pearson correlation between any pair of genes unchanged.*

**Proof.**  The per-gene map is $x_{g}^{\prime }=a_{g}+b_{g}\;\left(x_{g}-\mathrm{ctrl}\_{\mathrm{mean}}_{g}\right)$ with $a_{g}={\mathrm{mean}}_{g}$ and $b_{g}=\sqrt{{\mathrm{var}}_{g}/\mathrm{ctrl}\_{\mathrm{var}}_{g}}\geq 0$. Hence $E\left[x_{g}^{\prime }\right]=a_{g}={\mathrm{mean}}_{g}$ and $\mathrm{Var}\left[x_{g}^{\prime }\right]=b_{g}^{2}\;\mathrm{ctrl}\_{\mathrm{var}}_{g}={\mathrm{var}}_{g}$. For genes $g\neq g^{\prime }$, $\mathrm{Cov}\left(x_{g}^{\prime },x_{g^{\prime }}^{\prime }\right)=b_{g}b_{g^{\prime }}\;\mathrm{Cov}\left(x_{g},x_{g^{\prime }}\right)$, and dividing by $\sqrt{\mathrm{Var}\left[x_{g}^{\prime }\right]\mathrm{Var}\left[x_{g^{\prime }}^{\prime }\right]}=b_{g}b_{g^{\prime }}\sqrt{\mathrm{ctrl}\_{\mathrm{var}}_{g}\;\mathrm{ctrl}\_{\mathrm{var}}_{g^{\prime }}}$ cancels the positive factors $b_{g},b_{g^{\prime }}$, so the correlation is unchanged.    □

**Proposition** **2** (Leakage-freeness)**.** *AMM-SimWMag is a measurable function of the LOCO-admissible inputs $A_{h}$ only; it never reads the held-out stimulated cells $S_{h}$.*

**Proof.**  The magnitude scalar (part (a)) is fit on other types’ baselines and deltas and evaluated at *h* through $\mu_{h}^{c}$; the similarity weights (part (a′)) use only control profiles $\left\{\mu_{t}^{c}\right\}\cup \left\{\mu_{h}^{c}\right\}$; the predicted variance comes from Diagnostic (2), a function of $v_{h}^{c}$ and the other groups; and the transport of Equation (5) affine-rescales $C_{h}$. Each ingredient is a function of $A_{h}$, so their composition is; $S_{h}$ enters only the scoring step, identically for every method.    □

**Remark** **1** (Complexity and relation to optimal transport)**.** *All steps are closed-form and CPU-only: forming the deltas and similarity weights is O(KG) for K training groups, and the transport is $O(n_{h}G)$, with no iterative training. Per gene, matching the first two moments by a monotone affine map is exactly the closed-form Monge optimal-transport map between two one-dimensional Gaussians; AMM-SimWMag is thus a coordinatewise (diagonal) Gaussian OT calibration—a closed-form analogue of the second-moment alignment in CORAL [38] and of the learned neural transport in CellOT [12] and moscot [13].*

**Figure 1:**
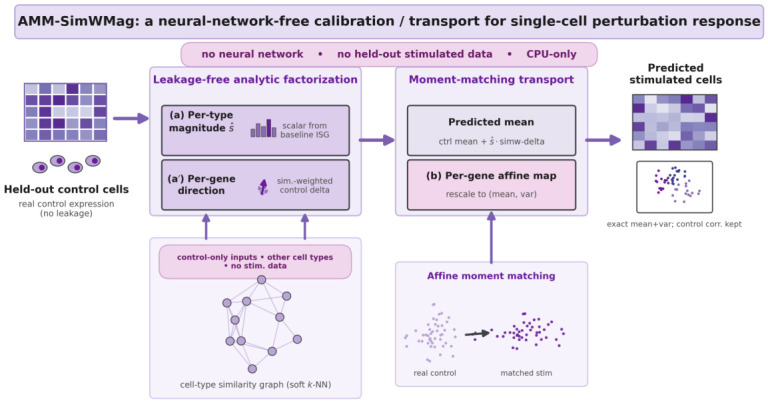
The AMM-SimWMag pipeline (no neural network). The real held-out control cells are mapped to predicted stimulated cells by (**a**) a per-type magnitude scale, (**a′**) a control-only similarity-weighted direction, and (**b**) a per-gene affine moment-matching step; every step is leakage-free and deterministic.

AMM-SimWMag combines (a′) and (b). Because (b) is a calibration layer over *any* mean predictor, we also report AMM-scGen (the same distribution step over scGen’s mean) to isolate whether a deep mean adds anything. For reference, the method names used throughout are: LinearDelta (the one-line mean shift, no calibration); -Mag (adds magnitude calibration); -SimWMag (adds similarity-weighted direction with magnitude); AMM- (adds the affine moment-matching distribution step); and AMM-scGen (the AMM step applied over scGen’s deep mean). “(no NN)” marks methods that use no neural network. Figure 1 summarises the pipeline.

## 6. Results

### 6.1. Single-Dataset Panel (Kang IFN-β, 7 Types × 3 Seeds)

**Figure 2:**
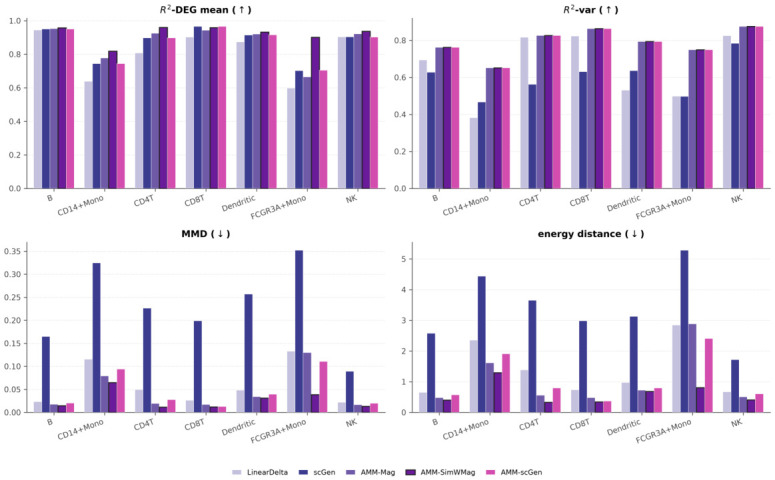
Per-held-out-type Kang results (7 held-out types, 3 seeds each). AMM-SimWMag and AMM-Mag match scGen on the mean (R^2^-DEG, top-left) and dominate it on the distribution metrics (R^2^-var, MMD, energy), with the largest distribution gains on the strong responders (CD14^+^/FCGR3A^+^ monocytes, dendritic cells).

Table 1 reports the full nine-metric panel. AMM-SimWMag is ≥scGen on *every* metric, with no neural network: it beats scGen on the mean (R^2^-DEG 0.924 vs. 0.868), wins deg_lfc_r (0.937 vs. 0.922), and wins all four distribution metrics (R^2^-var 0.789 vs. 0.601; MMD 0.027 vs. 0.230, 8×; energy 0.61 vs. 3.40, 5.5×; sliced-W 0.032 vs. 0.081). The deep AMM-scGen also reaches 9/9 here but adds nothing the calibration does not already provide. The gains hold on *every* held-out type and are largest on strong responders (Figure 2).

### 6.2. Cross-Dataset Generalisation (Four Datasets, Two LOCO Axes)

To test that this is not a Kang-specific artifact, we re-ran the identical panel on three further datasets: two intestinal infection datasets built from the Haber atlas [34] (Control vs. *Salmonella*; Control vs. *H. polygyrus*; eight cell types each, holding out a cell type) and a cross-species dataset from Hagai [35] (bone-marrow phagocytes, unstimulated vs. LPS 6 h, holding out a whole *species:* rat, rabbit, mouse, pig—scGen’s own cross-species experiment; 77,642 cells × 6000 genes). Table 2 counts how many of the nine metrics each method is better-or-equal to scGen on (within a 0.003 tolerance; ties are counted as wins, so each count upper-bounds the number of strict wins).

AMM-SimWMag is competitive-or-best on **8–9/9 metrics on every dataset.** It wins all four absolute distribution metrics on all four datasets (MMD/energy by 3–6×), beats scGen on the mean on all four (R^2^-DEG 0.924/0.887/0.888/0.576 vs. 0.868/0.742/0.846/0.409), and closes the per-gene direction gap (deg_lfc_r 0.937/0.895/0.888/0.732 vs. 0.922/0.772/0.879/0.715: tie-or-better everywhere, a significant win on *Salmonella*). In contrast, the deep AMM-scGen is *erratic* (9/9, 6/9, 5/9, 9/9): on *Salmonella*/*Hpoly*, scGen’s encoder underperforms the calibrated linear mean, and on Hagai its distribution metrics are so poor that moment matching alone wins. The robust contribution is therefore the calibration, not the network.

**Figure 3:**
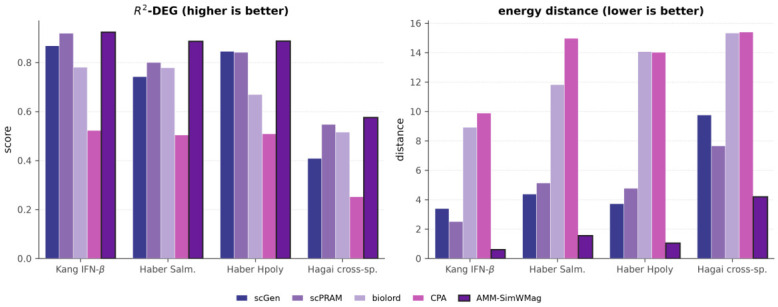
Per-dataset comparison on a representative location metric (R^2^-DEG, higher is better) and a representative distribution metric (energy distance, lower is better), for scGen and the three modern deep baselines vs. the no-NN AMM-SimWMag (black outline). The calibration is the only method that is strong on *both* axes across *all four* datasets, whereas each deep model degrades on at least one biology. Values are means over held-out groups and seeds.

**Honest cross-species nuance.** On *individual* Hagai species (Figure 3), scGen is more competitive on the mean/direction (e.g., rat: R^2^-mean 0.901 vs. 0.887, deg_lfc_r 0.794 vs. 0.739), but it is highly unstable across species (R^2^-mean 0.840±0.060, R^2^-DEG 0.409±0.192) while the calibration is steady (0.850±0.022, 0.576±0.086). Averaged over the four species the no-NN calibration wins all nine; we do not hide that scGen can lead on a single species. Because Hagai contributes only n=4 held-out groups (four held-out species, seeds averaged), its per-dataset test is severely underpowered—the smallest attainable two-sided signed-rank *p* is $2/2^{4}=0.125$, already above 0.05—so for this dataset we read the matched-pairs effect sizes as primary evidence and the *p*-values as secondary.

### 6.3. Statistical Significance

The wins/9 counts are point estimates. To test them, we use a two-sided Wilcoxon signed-rank test [39] of AMM-SimWMag vs. scGen, the unit of analysis being a held-out group within a dataset (seeds averaged). Each per-dataset test uses scipy’s zero_method="wilcox": held-out groups with a zero paired difference are dropped before ranking, and tied absolute differences receive average ranks; the exact null distribution is used when the number of non-zero pairs is ≤25 and the normal approximation otherwise, and all *p*-values are two-sided. We summarise each dataset by a *signed z*, defined as $\mathrm{sign}\left(\mathrm{median}\;\mathrm{paired}\;\mathrm{difference}\right)\cdot |\Phi^{-1}\left(p/2\right)|$, so that a positive value indicates AMM-SimWMag is favoured while the *p*-value stays two-sided. Because the four datasets differ in scale, difficulty and biology—and groups within a dataset share training data—the 27 held-out groups are *not* exchangeable, so a single pool of all 27 is not the right primary test. We therefore report three nested views, fixing the multiple-comparison family to the nine metrics of this single AMM-SimWMag-vs-scGen comparison and applying the Holm–Bonferroni correction [40] within it.

*Primary (per dataset).* Within each dataset, we test over its held-out groups (n=7/8/8/4 for Kang/Salmonella/Hpoly/Hagai; the minimum two-sided *p* at *n* is bounded below by $2/2^{n}$, so small datasets cannot reach significance alone). The effect favours AMM-SimWMag in all four datasets on seven of nine metrics, and matched-pairs rank-biserial effect sizes are large and positive (typically 0.7–1.0); full per-dataset tables are in results/significance_summary_stratified.md.

*Headline (dataset-stratified combination).* We combine the four datasets’ per-dataset signed *z*-scores with Stouffer’s method [41,42] weighted by $\sqrt{n_{\mathrm{dataset}}}$ (Table 3). This respects the grouping and assumes only a common *sign* of effect, not a common effect size. The $\sqrt{n_{\mathrm{dataset}}}$ weight is the standard weighted-Stouffer choice that maximises power when each dataset’s evidence scales with its number of held-out groups [42], up-weighting datasets that contribute more independent LOCO folds; because it could in principle drive the combination, we also report an equal-weight combination and two further robustness checks below. The calibration is significant on **8 of 9 metrics** after Holm correction (adjusted $p\leq 7.4\times 10^{-3}$), the effect favouring it in 4/4 datasets on seven metrics. The lone exception is deg_lfc_r.

*Pooled (descriptive).* For continuity with the conference-style analysis, naively pooling all 27 groups gives the same conclusion (significant on 8/9 metrics); we report each metric’s pooled median paired difference with a percentile-bootstrap 95% CI in the same source file, and treat it as descriptive only because the pooled units are non-exchangeable.

**Figure 4:**
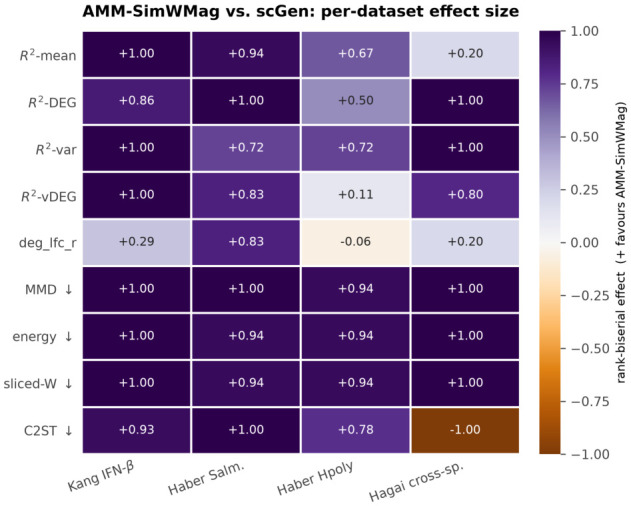
Matched-pairs rank-biserial effect size of AMM-SimWMag vs. scGen for each of the nine metrics on each of the four datasets (positive, purple, favours the calibration; sign convention oriented so that “better” is positive for all metrics). For the saturated metrics, ±1 reflects a negligible tied difference rather than a meaningful gap—e.g., the lone orange cell (C2ST on Hagai) arises because both methods sit at the ≈1.0 separability ceiling.

The only non-significant metric is deg_lfc_r: SimWMag is tie-or-better on every dataset (a significant per-dataset win on *Salmonella*, p=0.039, rank-biserial +0.83; ties on Kang/Hpoly/Hagai), favoured in 3/4 datasets, but combined it is a positive trend rather than a significant reversal of scGen’s last edge. We state this precisely rather than claiming 9/9. Figure 4 visualises the per-dataset matched-pairs rank-biserial effect sizes underlying Table 3: the effect favours AMM-SimWMag in almost every dataset × metric cell, the salient exceptions being the deg_lfc_r tie on Hpoly and the saturated C2ST on the Hagai dataset (both methods at the ≈1.0 ceiling).

### 6.4. Robustness of the Combination

Because the headline test up-weights larger datasets, we verify that the conclusion does not depend on that choice with three additional analyses (Table 4; full numbers in results/reviewer_robustness_summary.md). *(i) Equal-weight Stouffer.* Combining the four per-dataset signed *z*-scores with equal weights gives essentially the same result—significant on the same eight of nine metrics after Holm correction, deg_lfc_r again the lone exception—so the $\sqrt{n_{\mathrm{dataset}}}$ weighting is not driving the outcome. *(ii) Dataset-level sign test.* Treating each dataset as a single ± vote for whether its median paired difference favours AMM-SimWMag gives 4/4 in the same direction for seven metrics (two-sided sign-test p=0.125, the floor at four datasets) and 3/4 for deg_lfc_r and C2ST; the combination therefore never reverses sign across datasets. *(iii) Two-level (dataset, then group) bootstrap.* Resampling datasets and then held-out groups within each dataset ($10^{4}$ replicates) yields 95% confidence intervals for the mean paired difference that exclude zero for every metric except deg_lfc_r and R^2^-mean, and the probability that the effect favours AMM-SimWMag is ≥0.99 for seven metrics, 0.931 for R^2^-mean and 0.886 for deg_lfc_r. All three checks agree with the headline test. We read these checks as evidence that the *direction* of the effect is stable across these four benchmarks rather than as a proof of universal robustness: the top level has only four dataset clusters, so the two-level bootstrap is coarse and its *p*-values and confidence intervals are likely optimistic, and the leave-one-group-out folds within a dataset share training data and are therefore positively correlated, so the independence assumption underlying these tests is mildly violated and the reported significance may be slightly overstated.

### 6.5. Comparison to Modern Published Baselines

A natural objection is that scGen (2019) is dated. We therefore ran three *modern* published perturbation response models on the *identical* leave-one-group-out protocol and the *identical* nine-metric scoring code, so there is no protocol or scoring drift: scPRAM [8] (2024, attention + optimal transport over a VAE), biolord [9] (2024, disentangled deep generative model), and CPA [7] (2023, compositional perturbation autoencoder), each at the authors’ published default hyperparameters with no task-specific tuning. This is a deliberate *fairness floor:* we hand-tune neither the competitors nor the calibration, so the comparison reflects out-of-the-box behaviour on this transfer task rather than a tuned ceiling for any method. We exclude GEARS [14], which predicts *unseen genetic* perturbations from a gene–gene knowledge graph—a different task that does not fit our same-stimulus, unseen-group transfer. Table 5 counts each baseline’s wins/9 vs. scGen per dataset; Table 6 summarises the pooled paired significance of AMM-SimWMag vs. each.

Two findings stand out. First, *no single deep model is robust across biologies.* scPRAM is the strongest modern baseline—9/9 vs. scGen on Kang and Hagai, and the only method that beats *us* on any metric (Kang R^2^-var-DEG, 0.700 vs. 0.563)—yet it collapses on the two gut datasets (3/9, 0/9). biolord and CPA are weak throughout; CPA, which is built for combinatorial/dose screens with many shared conditions, is the weakest on this single-stimulus transfer task (pooled deg_lfc_r ≈0, often negative per fold; C2ST =1.0). Second, the no-neural-net AMM-SimWMag is a *statistically significant* improvement over every modern baseline pooled over the 27 held-out groups: 7/9 vs. scPRAM (the two exceptions, R^2^-var-DEG and deg_lfc_r, are non-significant ties), and 9/9 vs. both biolord and CPA; these vs.-baseline tests use the pooled Wilcoxon, and given the per-dataset wins of Table 5 the dataset-stratified conclusion is the same or stronger. For provenance, scGen was trained to its standard 150 epochs and CPA to its default 1200 epochs (all 81 folds), and scPRAM and biolord used their packaged defaults; we report every model at face value. CPA’s weakness on this single-stimulus LOCO task is expected from its design target (combinatorial/dose screens) and we do not down-weight it. Accordingly, “weakest” here means *at default settings on this transfer task*, not in general. Table 2 and Table 5 together summarise the robustness picture (Figure 3): the no-NN calibrations are uniformly high (8–9/9) across all four biologies, whereas the deep models—scPRAM most strikingly—swing between 9/9 and 0/9 depending on the dataset.

### 6.6. Biological Validation: Recovery of Canonical Interferon-Stimulated Genes

**Figure 5:**
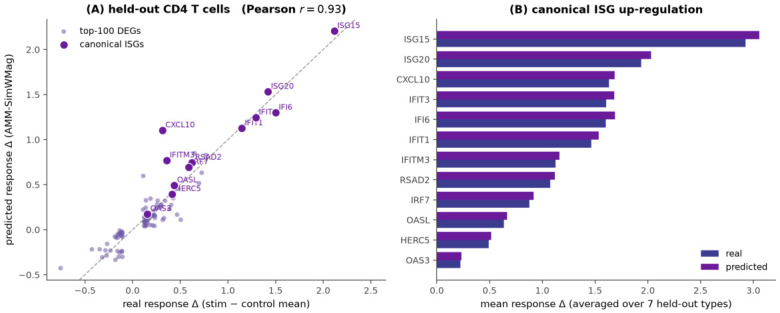
The leakage-free prediction recovers the canonical interferon response. (**A**) Predicted vs. real per-gene response Δ (stimulated minus control mean) over the top-100 DEGs of held-out CD4 T cells; canonical ISGs (highlighted) lie on the diagonal (Pearson r=0.93). (**B**) Real vs. predicted mean response Δ for canonical ISGs, averaged over all seven held-out PBMC types; the predicted up-regulation tracks the measured one gene by gene. No held-out stimulated cell enters any prediction.

The aggregate metrics above say the predicted response is accurate; we now ask whether it is accurate *on the right genes*. For each held-out PBMC type, we compared the predicted per-gene response $\Delta ={\hat{\mu }}_{h}-\mu_{h}^{c}$ with the real one $\mu_{h}^{s}-\mu_{h}^{c}$, focusing on the canonical interferon-stimulated genes that define the IFN-β programme [2]. Figure 5A shows that for held-out CD4 T cells the predicted and real response deltas agree closely over the top 100 differentially expressed genes (Pearson r=0.93, the same quantity as the deg_lfc_r metric of Table 1, whose mean over folds is 0.937), and that the textbook ISGs—*ISG15*, *ISG20*, *IFI6*, *IFIT1*, *IFIT3*, *IFITM3*, *OASL*, *RSAD2*, *CXCL10*, *IRF7*—are exactly the strongly up-regulated genes the calibration predicts, with no held-out stimulated cell used to produce the prediction (Proposition 2). Averaged over all seven held-out types (Figure 5B), the predicted ISG up-regulation tracks the measured magnitude gene by gene, recovering the known ordering (*ISG15* and *ISG20* largest). Because the prediction for a held-out type is built only from the other types’ responses and that type’s own control cells, this is direct evidence that the conserved IFN-β direction—not type-specific memorisation—is what the similarity-weighted factor transfers, consistent with the cross-species conservation of the core ISG response [3,35].

To confirm that the recovered response is enriched for the *right* biology rather than merely correlating gene by gene, we ran an offline hypergeometric over-representation (GSEA-style) test of the top 100 predicted up-regulated genes against the 50 MSigDB Hallmark gene sets, using the 6998 Kang genes as background. A total of 12 of the 50 pathways are enriched at FDR < 0.05, and the top hits are exactly the expected interferon and inflammatory programmes: Interferon Gamma Response (overlap 34/92, $p=3.6\times 10^{-41}$, $\mathrm{FDR}=1.8\times 10^{-39}$), Interferon Alpha Response (22/39, $p=9.3\times 10^{-32}$, $\mathrm{FDR}=2.3\times 10^{-30}$), Inflammatory Response (13/81, $\mathrm{FDR}=1.3\times 10^{-9}$) and TNF-α Signaling via NF-κB (12/104, $\mathrm{FDR}=2.7\times 10^{-7}$); full results are in results/gsea_validation_summary.md. We note that although the Kang stimulus is IFN-β, the induced interferon-stimulated genes are largely shared across type-I (IFN-α/β) and type-II (IFN-γ) interferon signalling, so the enrichment of both the alpha- and gamma-response Hallmark sets is expected; conversely, this overlap means the prediction reflects a generic ISG programme and cannot by itself attribute the response to IFN-β specifically.

## 7. Discussion

Our central finding is that scGen’s advantage on LOCO perturbation transfer decomposes into two leakage-free analytic factors and can be recovered without any neural network, a constructive counterpart to a growing body of critical benchmarking. Ahlmann-Eltze et al. [21] report that foundation and deep models do not consistently beat linear or mean baselines for genetic perturbation effects; on the cell-type/species transfer task, we go one step further and localise *where* the deep edge comes from (per-type magnitude and per-gene direction), then show that supplying those two quantities analytically closes the gap. This is not evidence that deep models are useless; rather, on this task their useful signal is low-dimensional and an explicit, inspectable calibration captures it.

This reframes the role of a method like AMM-SimWMag. It is best read as a *strong, CPU-only baseline:* before a new model’s added complexity is judged worthwhile on this task, it should have to beat the calibration. That bar applies to foundation models such as scGPT [17] and scFoundation [18], and to optimal-transport approaches such as CellOT [12]. Because the calibration is deterministic and leakage-audited, it also separates how much of a deep model’s reported gain is genuinely learned from how much merely reflects magnitude and direction effects that a calibration already supplies.

Our evaluation also argues for two methodological habits. First, distributional agreement should be reported with several complementary measures, because a single metric (e.g., the headline R^2^) hides the distribution-level differences where calibration helps most; we use MMD, energy distance (as popularised for perturbations by scPerturb [28]), and sliced-Wasserstein. Second, significance on multi-dataset benchmarks should respect dataset grouping. Our dataset-stratified Stouffer test and rank-biserial effect sizes give a more honest picture than naively pooling non-exchangeable held-out groups.

**Biological reading of the two factors.** The decomposition is not only a statistical convenience; it mirrors the biology of the benchmarks. The per-gene *direction* corresponds to which genes go up or down under the stimulus—a largely conserved programme, e.g., the canonical interferon-stimulated genes induced by IFN-β [2] or the core LPS response shared across mammals [3]—whereas the per-type (or per-species) *magnitude* captures how strongly a given cell type or species mounts that conserved programme. This is consistent with the calibration’s distribution-level gains being largest on the strong responders (CD14^+^/FCGR3A^+^ monocytes and dendritic cells in Kang; Figure 2) and with a single magnitude scalar transferring cleanly across the held-out species in Hagai: the response *shape* is shared, and only its *scale* needs to be set per group.

### Limitations

We close with the limitations and honest caveats that bound these claims:

- **The framing is inverted.** It is common that a bigger deep model beats simple baselines. Here, the reverse holds: a no-neural-net calibration matches or beats the deep state of the art, because scGen’s edge decomposes into a leak-free magnitude scalar and a leak-free direction.
- **C2ST is not won by anyone** (≈0.99 for all methods). The diagnostics (Section 4) show this follows from the sparse, non-Gaussian, gene-correlated structure of scRNA-seq under LOCO for the methods and oracle approximations we evaluated (even a true-moment Gaussian and a true-marginal hurdle oracle stay near 1.0), rather than being a proof that no generator could ever win or a failure of our method.
- **deg_lfc_r is a tie, not a win,** once tested rigorously (Section 6.3).
- **The calibration is a transport, not a sampler.** Because the distribution step affine-rescales the *real* held-out control cells (Section 5), the predicted cells inherit the control population’s per-cell structure and add no new stochasticity. This is exactly why the method wins the distribution *distances* yet cannot win C2ST, and it limits sample diversity for downstream uses that need freshly synthesised cells rather than a calibrated transport of controls.
- **No single deep baseline is robust across biologies.** On the same protocol, scPRAM is excellent on Kang/Hagai yet collapses on the gut datasets (Hpoly 0/9 vs. scGen), and CPA (designed for combinatorial/dose screens with shared conditions) is weakest at its default settings on this single-stimulus transfer (deg_lfc_r ≈ 0, C2ST = 1.0). A fixed analytic calibration is more dependable here than any one published network (Section 6.5).
- **Scope.** We evaluate single-perturbation LOCO transfer on log-normalised HVG expression. Combinatorial/dose perturbations [7,11], genetic Perturb-seq screens [14,24,25,26], count-space likelihoods, and trajectory effects are out of scope; the affine map assumes an approximately affine control→stim relationship per gene, which holds well on these datasets but need not hold universally.
- **Linear response, not nonlinear cell-fate change.** The calibration models the response as a per-gene affine rescaling of the control population, so it captures graded up-/down-regulation of an existing programme but not strongly nonlinear effects—switch-like activation, cell-fate or cell-state transitions, or the appearance of qualitatively new sub-populations. On perturbations that reprogramme cells rather than modulate a conserved response, a learned nonlinear model may be necessary and the affine assumption is expected to break down.
- **One fixed configuration across all datasets.** We deliberately run AMM-SimWMag with the *same* hyperparameters (top-100 DEGs, variance floor $\varepsilon =10^{-6}$, softmax similarity temperature, seeds 0,1,2) on every dataset to avoid per-dataset tuning. This makes the comparison fair and the method turnkey, but it also means we do not report a per-dataset-optimised ceiling; a dataset-specific configuration could do better still, and conversely the single setting may be suboptimal on a biology unlike the four evaluated here.
- **Benchmark data, not clinical data.** The deep baselines we compare against are designed for, and intended to scale to, larger and more complex clinical or patient-derived datasets; our four datasets are curated research benchmarks. A strong result here does not establish that a fixed analytic calibration would remain competitive on the larger, noisier and more heterogeneous data those models target, and we do not claim clinical applicability.
- **The IFN-β label denotes the experimental stimulus, not a molecular attribution.** The Kang benchmark is stimulated with IFN-β, but what the calibration recovers is the shared type-I (IFN-α/β) and type-II (IFN-γ) interferon-stimulated-gene (ISG) programme—the pathway-enrichment (GSEA) analysis lights up both the alpha- and gamma-response Hallmark sets. The prediction therefore cannot by itself attribute the response to IFN-β specifically, because other interferons drive a largely overlapping ISG programme and could produce a similar signature.

## 8. Conclusions

On the leave-one-group-out perturbation-transfer task, the advantage of the standard deep baseline scGen over a one-line linear shift is small, low-dimensional and, crucially, learnable without a neural network. By decomposing that advantage into a leakage-free per-type magnitude and a leakage-free per-gene direction, and adding a per-gene affine moment-matching step, our calibration AMM-SimWMag is competitive-or-best against scGen on 8–9 of 9 metrics across 4 datasets spanning 3 biologies and 2 LOCO axes, is significant on 8 of 9 metrics under a dataset-stratified test, and beats the modern baselines scPRAM, biolord and CPA pooled, while staying CPU-only and fully reproducible. We deliberately report the two places it does *not* win (C2ST, which saturates near 1.0 for every method evaluated here and for oracle moment- and hurdle-based approximations, and the per-gene log-fold-change correlation, a statistical tie). We therefore recommend AMM-SimWMag as a cheap, transparent baseline that new deep and foundation models for this task should be required to surpass, and we encourage multi-metric, dataset-stratified evaluation with reported effect sizes as the default for this benchmark.

## 9. Reproducibility

All calibration methods, diagnostics, and statistics are CPU-only; only scGen requires a GPU. The complete analysis code, the processed Kang dataset, and the scripts to rebuild the other datasets are provided as Supplementary Materials with this submission; the exact shell commands that regenerate every table and figure are listed in the accompanying README. All runs use fixed seeds 0,1,2 (each Hagai species is scored as one held-out group, with its three seeds averaged), and the extra datasets are built with data_processing_make_haber.py and data_processing_make_hagai.py. Package versions (scanpy, scvi-tools/cpa/scpram/biolord, scikit-learn, scipy), per-dataset data provenance, and run-times are recorded in the chronological hypothesis→result log EXPERIMENT_LOG.md; full tables are in experiments/results/. Every figure and table is regenerated from those summary files by the listed scripts, so no figure is hand-edited.

### Computational Cost

  Because a central claim of this work is efficiency, we measured wall-clock time and peak resident-memory increment directly on the Kang benchmark (16,893 cells ×6998 genes, 7 held-out PBMC types, seed 0) on 8 logical CPUs with no GPU (Table 7). AMM-SimWMag is timed on all seven held-out folds; scGen’s 150-epoch VAE training, which costs minutes per fold on CPU, is timed on two representative folds and reported as the per-fold cost, with the full-sweep figure obtained by multiplying the measured per-fold mean by seven. All numbers are CPU-only measurements; GPU time is not applicable because the entire study runs on CPU. On the same hardware, the neural-network-free calibration completes a held-out fold in 0.44 s vs. 674 s for scGen—an order-of-magnitude (≈$10^{3}\times$; per fold ≈1500×) speed-up—with a smaller peak-memory footprint and no gradient-based training. We emphasise that this ≈$10^{3}\times$ figure characterises a *CPU-only* deployment and is not a universal efficiency claim: the 674 s is the wall-clock of a forced-CPU, 150-epoch VAE training, which drops substantially on a GPU, so on GPU hardware the gap would narrow; we do not quote a GPU wall-clock because none was measured in this study. The reported scGen 674±50 s is a mean ± SD over only n=2 timed folds. The timed AMM-SimWMag covers the mean branch (steps (a)/(a′)) only; the per-gene variance affine step (b) has cost $O(n_{h}G)$, negligible relative to the mean branch, and does not change the order of magnitude.

## Figures and Tables

**Table 1:** Kang IFN-β, leave-one-cell-type-out (7 types × 3 seeds), mean over folds. Higher is better for R^2^ metrics and deg_lfc_r; lower is better for MMD, energy, sliced-W, C2ST. “no NN” methods use no neural network. R^2^-vDEG abbreviates R^2^-var-DEG (variance restricted to DEGs). Bold values mark the best score in each column (ties included; ↓ = lower is better); the proposed method (AMM-SimWMag) is set in bold in the row label.

Model	R^2^-Mean	R^2^-DEG	R^2^-Var	R^2^-vDEG	deg_lfc_r	MMD↓	Energy↓	Sliced-W↓	C2ST↓
LinearDelta	0.903	0.810	0.653	0.277	0.896	0.060	1.374	0.045	0.997
LinearDelta-Mag (no NN)	0.933	0.872	0.653	0.277	0.896	0.044	0.991	0.038	0.997
scGen (deep)	0.933	0.868	0.601	0.349	0.922	0.230	3.396	0.081	0.998
LinearDelta-SimWMag (no NN)	**0.961**	**0.924**	0.653	0.277	**0.938**	**0.026**	**0.577**	**0.030**	0.996
AMM-Mag (no NN)	0.933	0.872	**0.789**	**0.563**	0.896	0.045	1.036	0.040	0.998
**AMM-SimWMag (no NN)**	0.960	**0.924**	**0.789**	**0.563**	0.937	0.027	0.612	0.032	0.997
AMM-scGen (deep)	0.932	0.869	**0.789**	**0.563**	0.922	0.046	1.064	0.040	**0.994**

**Table 2:** Wins/9 vs. scGen per dataset (better-or-equal within tol. 0.003).

Method (vs. scGen)	Kang IFN-β	Haber *Salm.*	Haber *Hpoly*	Hagai Cross-sp.	Worst
LinearDelta-SimWMag (no NN)	8/9	9/9	8/9	8/9	8/9
**AMM-SimWMag (no NN)**	9/9	9/9	8/9	9/9	**8/9**
AMM-Mag (no NN)	8/9	9/9	7/9	9/9	7/9
AMM-scGen (deep)	9/9	6/9	5/9	9/9	5/9

**Table 3:** Dataset-stratified significance of AMM-SimWMag vs. scGen (headline test): Stouffer combination of the four per-dataset two-sided Wilcoxon signed-rank *z*-scores, weighted by $\sqrt{n_{\mathrm{dataset}}}$. Combined *p* and Holm-adjusted *p* across the nine metrics; “datasets +” = number of the four datasets whose median paired difference favours AMM-SimWMag.

Metric	Combined *Z*	Combined *p*	Holm *p*	Datasets +
R^2^-mean	+3.48	$5.0\times 10^{-4}$	$1.5\times 10^{-3}$	4/4
R^2^-DEG	+3.68	$2.4\times 10^{-4}$	$1.2\times 10^{-3}$	4/4
R^2^-var	+3.74	$1.9\times 10^{-4}$	$1.1\times 10^{-3}$	4/4
R^2^-var-DEG	+2.90	$3.7\times 10^{-3}$	$7.4\times 10^{-3}$	4/4
deg_lfc_r	+1.43	$1.5\times 10^{-1}$	$1.5\times 10^{-1}$ (n.s.)	3/4
MMD	+4.59	$4.5\times 10^{-6}$	$4.1\times 10^{-5}$	4/4
energy	+4.45	$8.5\times 10^{-6}$	$6.8\times 10^{-5}$	4/4
sliced-W	+4.45	$8.5\times 10^{-6}$	$6.8\times 10^{-5}$	4/4
C2ST	+3.59	$3.3\times 10^{-4}$	$1.3\times 10^{-3}$	3/4

**Table 4:** Robustness of the dataset-stratified combination. sqrt-*nZ* and equal-weight *Z* are the two Stouffer combinations of the per-dataset signed Wilcoxon *z*-scores; “sign” counts how many of the four datasets favour AMM-SimWMag (two-sided sign-test *p*); the two-level bootstrap gives the 95% CI of the mean paired difference and P(effectfavoursAMM-SimWMag).

Metric	Sqrt-*n Z*	Equal-Wt *Z*	Equal-Wt Holm *p*	Sign (4)	Bootstrap 95% CI	P(AMM)
R^2^-mean	+3.48	+3.30	$3.0\times 10^{-3}$	4/4 (0.125)	[−0.005,+0.036]	0.931
R^2^-DEG	+3.68	+3.67	$1.2\times 10^{-3}$	4/4 (0.125)	[+0.020,+0.172]	1.000
R^2^-var	+3.74	+3.74	$1.1\times 10^{-3}$	4/4 (0.125)	[+0.068,+0.240]	1.000
R^2^-var-DEG	+2.90	+2.91	$7.1\times 10^{-3}$	4/4 (0.125)	[+0.031,+0.237]	0.995
deg_lfc_r	+1.43	+1.35	$1.8\times 10^{-1}$ (n.s.)	3/4 (0.625)	[−0.012,+0.082]	0.886
MMD	+4.59	+4.51	$5.7\times 10^{-5}$	4/4 (0.125)	[+0.120,+0.228]	1.000
energy	+4.45	+4.39	$8.9\times 10^{-5}$	4/4 (0.125)	[+2.509,+5.195]	1.000
sliced-W	+4.45	+4.39	$8.9\times 10^{-5}$	4/4 (0.125)	[+0.048,+0.082]	1.000
C2ST	+3.59	+3.37	$3.0\times 10^{-3}$	3/4 (0.625)	[+0.001,+0.068]	0.994

**Table 5:** Modern published baselines: wins/9 vs. scGen per dataset (better-or-equal within tol. 0.003). AMM-SimWMag (no neural network) shown for reference.

Method (vs. scGen)	Kang IFN-β	Haber *Salm.*	Haber *Hpoly*	Hagai Cross-sp.
scPRAM (2024)	9/9	3/9	0/9	9/9
biolord (2024)	1/9	2/9	0/9	3/9
CPA (2023)	1/9	0/9	0/9	1/9
**AMM-SimWMag (no NN)**	9/9	9/9	8/9	9/9

**Table 6:** Pooled paired significance of AMM-SimWMag vs. each modern baseline over n=27 held-out groups (two-sided Wilcoxon, α=0.05). Metrics on which the calibration wins all 27 groups attain the exact n=27 floor $2/2^{27}\approx 1.5\times 10^{-8}$.

AMM-SimWMag vs.	Sig. Better/9	Metrics It Does *Not* Beat
scPRAM (2024)	7/9	R^2^-var-DEG, deg_lfc_r (n.s. ties)
biolord (2024)	9/9	—
CPA (2023)	9/9	—

**Table 7:** Measured CPU time and peak memory on the Kang benchmark (7 held-out types, 16,893×6998, seed 0; 8 logical CPUs, no GPU). scGen per-fold time is the mean ± SD of two timed folds and the full sweep is that mean ×7 (over n=2 timed folds); AMM-SimWMag is measured on all seven folds and its timing covers the mean branch (steps (a)/(a′)) only.

Method	Per-Fold Time	Full Sweep (7 Folds)	GPU Time	Peak Memory
AMM-SimWMag (no NN)	0.44±0.05 s	3.1 s (measured)	not used (CPU-only)	+705 MB
scGen (VAE, 150 epochs)	674±50 s	≈4720 s (est.)	not used (CPU-only)	+909 MB

## Data Availability

All datasets analysed in this study are publicly available from their original publications: the Kang IFN-β PBMC dataset [33], the Haber small-intestinal atlas [34] (the *Salmonella* and *H. polygyrus* infection arms), and the Hagai cross-species dataset [35]. The complete analysis code, the processed Kang dataset, and the scripts to rebuild the other datasets are provided as Supplementary Materials with this submission and are available from the corresponding author on request.

## Supplementary Materials

The following supporting information can be downloaded at: https://www.mdpi.com/article/10.3390/genes17070816/s1.
